# A Rare Case of Diffuse Idiopathic Pulmonary Neuroendocrine Cell Hyperplasia

**DOI:** 10.7759/cureus.2525

**Published:** 2018-04-24

**Authors:** Andrea Anampa-Guzmán, Luis E Raez

**Affiliations:** 1 Faculty of Medicine, Universidad Nacional Mayor De San Marcos; 2 Medical Oncology and Hematology, Memorial Cancer Institute

**Keywords:** hispanic, thorax, carcinoma

## Abstract

We describe the case of a 53-year-old woman who visited many pneumologists without a diagnosis until finally being diagnosed with diffuse idiopathic pulmonary neuroendocrine cell hyperplasia (DIPNECH). It is a relatively new disease characterized by neuroendocrine cell hyperplasia in small airways. She has stable DIPNECH and neuroendocrine carcinoma with somatostatin therapy.

## Introduction

Diffuse idiopathic pulmonary neuroendocrine cell hyperplasia (DIPNECH) is a rare lung disease characterized by the World Health Organization (WHO) as a proliferation of scattered, single pulmonary neuroendocrine cells (PNECs), tumorlets, and/or a linear proliferations of these cells confined to the bronchial and bronchiolar epithelium. The definition is histological and does not include symptomatology. PNECs are present throughout the entire respiratory tract in the pulmonary epithelium. These cells contain calcitonin, chromogranin A, bombesin, and serotonin. The inappropriate and excessive production of these secretory products may lead to constrictive fibrotic lesions. The number of PNECs can increase in some chronic lung diseases. However, DIPNECH is seen as a primary neuroendocrine cell proliferation [1].

DIPNECH is considered a precursor lesion for carcinoid tumors, which can be typical or atypical carcinoids depending on their grade—low and intermediate grade, respectively [2]. However, the risk of developing a clinically significant disease from these lesions appears to be low. DIPNECH has not been related to small or large cell carcinomas. DIPNECH has been associated with bronchiectasis, fibrosis, and obliterative bronchiolitis [3].

DIPNECH is a relatively new disease; it was described in 1992 by Aguayo et al. It is a very rare disease and is commonly misdiagnosed. The common clinical presentation is a nonsmoker woman between the ages of 50 and 70 with a dry cough and/or dyspnea that slowly worsens over several years. However, patients can be asymptomatic and only find they have the disease when pulmonary nodules are detected in a radiological evaluation. The physical examination is usually nonspecific and pulmonary function tests in these patients are variable. They can be normal, obstructive, restricted, or a mix of restrictive and obstructive [4].

## Case presentation

We present the case of a white, Hispanic, 58-year-old, non-smoker female. She has a past medical history of obstructive sleep apnea and chronic obstructive pulmonary disease (chronic bronchitis). Her travel history includes the Dominican Republic and Caribbean Islands. She worked for several years in an automobile repair shop and was exposed to lead and paint. Her current occupation is as a telephone operator in a call center, which requires her to speak continually.

**Figure 1:**
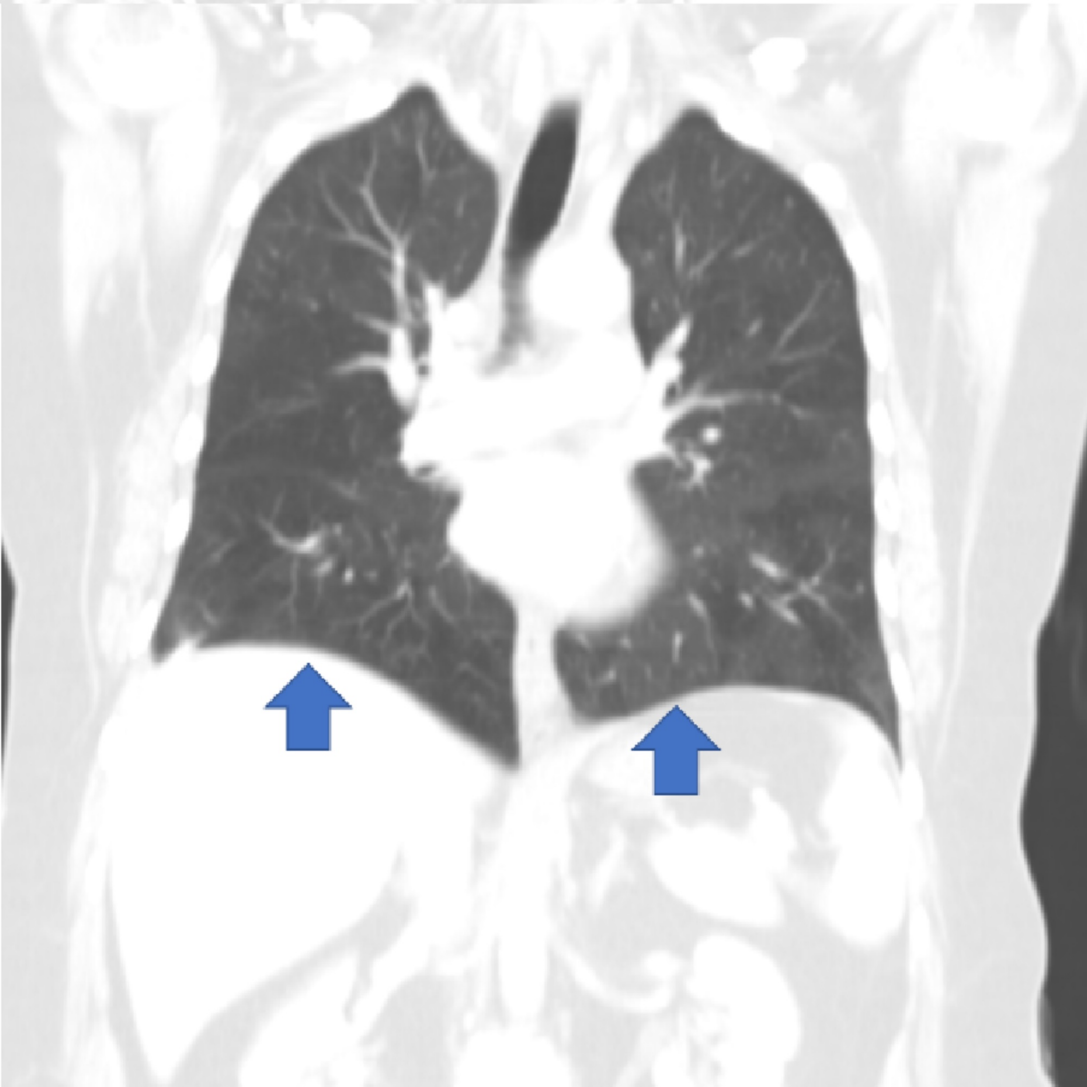
Mild ground glass infiltrates in the lung bases

Fifteen years ago, she developed a progressive cough. During the following four years, she was evaluated by more than eight pulmonologists who were unable to make a diagnosis. She developed a productive cough with white sputum and blood titer. Her alpha-1-antitrypsin serum, perinuclear anti-neutrophil cytoplasmic antibody (P-ANCA), cytoplasmic antineutrophil cytoplasmic antibody (C-ANCA), and rheumatoid factor were all within normal limits. Her purified protein derivative (PPD) and fungal infection tests were negative. Lung function tests revealed an obstructive pattern. Her forced expiratory volume in one second (FEV1)/forced vital capacity (FVC) was 70% and total lung capacity (TLC) was 72%. A computed tomography (CT) scan showed mild ground glass infiltrates in the lung bases (Figure 1).

Finally, in December 2011, a lung biopsy via assisted thoracoscopic surgery (VATS) was performed, and she was diagnosed with constrictive bronchiolitis and diffuse idiopathic neuroendocrine cell hyperplasia with carcinoid tumorlets. She was treated with octreotide; however, the treatment was interrupted several times due to issues with insurance and difficulties visiting the medical center. Her treatment was restarted in our hospital after three years in May 2014. Since then, CT scans taken every six months show a stable disease. She currently has a stable radiographic disease with no new complaints during the over two years of follow-up.

## Discussion

DIPNECH is a rare lung disease characterized by a marked increase in neuroendocrine (NE) cells. The pathological diagnosis criteria in surgical samples are the presence of five or more NE cells, either singly or in clusters, located within the basement membrane of the bronchiolar epithelium of at least three bronchioles, combined with three or more carcinoid tumorlets [1]. The NE cells in DIPNECH are positive for neuroendocrine markers of differentiation such as synaptophysin, chromogranin, and CD56 [2]. CD10 is stained consistently in neuroendocrine cells. On the other hand, the expression of bombesin, BCL-2, retinoblastoma protein, p27, and calcitonin is variable [5].

It is important to mention that DIPNECH has only been described in pathology, and there are no clinical or radiological criteria. DIPNECH has a predilection for non-smoking, middle-aged women and is associated with a predominantly obstructive ventilatory pattern in pulmonary function tests seen in obliterative bronchiolar fibrosis, similar to our patient. While it has been proposed that high clinical suspicion can be enough for the diagnosis, DIPNECH can also be asymptomatic and require further testing to diagnose [6].

The common imaging findings of DIPNECH are bilateral and diffuse airway abnormalities and pulmonary nodules. The airway abnormalities include mosaic attenuation with air trapping, diffuse bronchial wall thickening, mucoid impaction, and mild bronchiectasis. The presence of pulmonary nodules can be confused with metastatic cancer, but the nodules of DIPNECH nodules are typically noncalcified, rounded, and well-defined. The majority of the nodules are six mm in size and are predominately distributed in lower or midlung fields. In one cohort of 21 patients with an average length of follow-up of 1054 days, the number of nodules was stable in most subjects [7].

Biochemical testing includes serum chromogranin A, which is synthesized by PNECs. In patients with DIPNECH, there is an increased serum level of chromogranin A and treatment with somatostatin analogs reduces these levels. Another biochemical test is 5-hydroxyindoleacetic acid in a 24-hour urine collection. This test is not affected by treatment. DIPNECH is a rare condition and is frequently under-diagnosed or misdiagnosed. Common misdiagnoses of DIPNECH are metastatic diseases from a remote primary tumor and obstructive lung diseases, such as nonatopic asthma and chronic bronchitis [8].

According to the National Comprehensive Cancer Network [9], patients can be observed with chest CT scans without contrast every 12 months or for new symptoms. The treatment of DIPNECH remains unclear. Due to the slow progression and indolent nature of DIPNECH, conservative management is often used. There is evidence that somatostatin analogs, such as octreotide or lanreotide, alleviate the chronic cough. Treatment with glucocorticoids and surgical resection are avoided, if possible. However, a progressive disease with obliterative bronchiolitis requiring lung transplantation has been reported. The patients who receive transplant remain stable over many years, independent of the mode of presentation, although a few presented with severe airflow obstruction [10].

## Conclusions

DIPNECH is a relatively new disease characterized by neuroendocrine cell hyperplasia in small airways. Our patient visited different pulmonologists for years without a proper diagnosis. Even though DIPNECH is a rare disease, it should be included in the differential diagnosis of patients with obstructive respiratory symptoms and compatible radiological alterations, especially in women without a history of smoking.
